# Patients’ perception of service quality in a healthcare not-for-profit organisation

**DOI:** 10.4102/safp.v64i1.5490

**Published:** 2022-09-26

**Authors:** Melene Strauss, Renata Schoeman

**Affiliations:** 1 University of Stellenbosch Business School, Bellville, South Africa; 2 Department of Leadership, University of Stellenbosch Business School, Bellville, South Africa

**Keywords:** service, quality, healthcare, measurement, improvement, value, SERVPERF, not-for-profit

## Abstract

**Background:**

Service organisations should be aware of those elements that are perceived as excellent quality and incorporate these as part of their service offering. However, a not-for-profit (NPO) healthcare organisation consists of a diverse group of stakeholders who have different perspectives and interests. Service quality therefore requires a multidimensional definition that comprehends all their needs and expectations.

**Methods:**

Perceived service quality experienced by patients was measured by completion of the Service Performance (SERVPERF) questionnaire. A total of 111 patients completed the questionnaire across three mobile clinics supported by an NPO.

**Results:**

The research results suggested that service quality at the mobile clinics was of a very high standard, with no meaningful differences between clinics, age groups or gender. However, the responses had very little variance and could have been subjected to response bias or extreme bias. The absence of a comparator organisation could also have had an influence on responses given by respondents.

**Conclusion:**

Healthcare service organisations should strive towards maintaining high standards and engage in continuous measurement and improvement of their service quality as part of their quality management process. By measuring the current level of service experienced by patients, insights have been identified where adjustments might have a positive effect on perceived value. Future research recommendations include suggestions to increase the sample population, taking the service setting into account and further studies to confirm the validity and reliability of solicited service quality questionnaires in a NPO setting.

## Introduction

Service quality experience is context dependent; if you expect nothing, anything is good. Service quality is viewed as the summation of all stakeholders’ perceptions of the service that is delivered, and a customer’s opinion of the value of the product or service is one of the critical elements in a successful service delivery model.^1,2,3^ But service quality is often an intangible concept to measure.^4^ Furthermore, the subjectivity of perceptions makes it challenging to define and evaluate quality.^2,3,4^ Customer satisfaction is significant in medical care, and it is an essential part of the evaluation of the performance and quality of services provided by public organisations.^5^ Good quality healthcare can be defined as providing patients with the appropriate services in a technically competent manner. In contrast to manufactured goods, healthcare services are produced and expended at the same time and cannot be stored for later consumption, which means that healthcare service is less likely to have a final quality check. Therefore, quality control is difficult because the consumer cannot ascertain quality prior to purchase or consumption.^4^

Healthcare systems are globally developing healthcare reform measures that are value driven, moving away from being volume driven.^6,7^ The value-based healthcare concept is focussed on the outcome that matters to the patient, compared with the costs of treating the disease. Excellent medical care can be defined as a reflection of values and goals of a healthcare system and the society in which it exists.^8^ One of the indicators of excellent care is the outcome of medical care in terms of recovery, restoration of function and survival.^8^ Donabedian, father of quality assurance and poet, has been credited with developing an efficient healthcare framework that measures quality in terms of structure, process and outcome.^9^

Value for the patient is therefore achieved by receiving quality and effective care.^6^ The interests of all stakeholders can be unified through the attainment of better health outcomes or reducing healthcare costs.^7^ This reduction in costs is associated with favourable health outcomes that have been obtained most efficiently. The critical stakeholder in value-based healthcare is the patient, and he or she determines the value of the care received. Healthcare should thus be organised in such a way that a distinct set of patient needs is met, over the full cycle of care. Consequently, the aim is to improve health outcome with increased efficiency, and to achieve this, measurement of outcomes and costs is essential.^7^

Value for customers or consumers is often described as the monetary (i.e. price) or nonmonetary (i.e. time, effort or opportunity cost) sacrifice to acquire a beneficial component.^10,11,12,13^ Therefore, the perceived value for the customer is a result of intellectual integration of money and time sacrifices versus the experience of the service received.^14,15^ As a result, customer value is more significant than only an exchange between the quality and price of the service.^16^

Service quality is recognised as a precursor for customer satisfaction and behavioural intent.^1,13^ An improved level of service quality expands customer satisfaction and loyalty with a resultant positive effect on customer retention.^17^ Service quality is often measured in the service industry using various measurement tools available, based on the expectation and perception of quality expected and received. Customer assessment feedback on their expectations and priorities is necessary, as it can aid in the improvement of services.^18^ Customers who have had an experience of the service provided can effectively point to strengths and weaknesses in the system.^19^ A service organisation should therefore be aware of what elements their stakeholders perceive as excellent quality and incorporate these as part of their service delivery model.^20^ In a setting where profit is not a main quality driver, more insight is required into nonmonetary sacrifice and perception of service quality by the patient.

The aim of the study reported in this article was to evaluate perceived service quality experienced by patients in a not-for-profit (NPO) healthcare setting. Key objectives were to measure the current level of service quality from the patient’s perspective, to identify gaps in quality and to suggest recommendations for improvement. The diverse group of stakeholders of an NPO will have different perspectives and interests and require a multidimensional definition of service quality that comprehends all their needs and expectations.^4^ The multidimensional aspects of service quality identified during this study could therefore guide towards a more nuanced understanding whilst measuring service quality in different settings.

## Methods

### Study design, setting and sampling

Perceived service quality experienced by patients was evaluated by administering a cross-sectional quantitative questionnaire to patients attending a NPO healthcare mobile clinic.

The study population consisted of patients from three mobile clinics that are part of a pharmaceutical company’s corporate social responsibility (CSR) projects. The mobile clinics provide healthcare services to low-income communities. The cost of the service is subsidised by the pharmaceutical company, and patients pay reduced fees compared with private healthcare. Each clinic is managed by a nurse and aims to reduce common illnesses and noncommunicable chronic diseases. The mobile clinics therefore provided an appropriate setting to evaluate service quality in a nonprofit setting.

Convenience sampling was applied to select a study sample from patients who visited the clinics. Respondents had to meet the following criteria before completing a questionnaire:

- be 18 years or older
- be willing and able to provide informed consent
- be a patient who had received care at the mobile clinic where the questionnaire was being administered.

### Data collection

The Service Quality (SERVQUAL) questionnaire was designed to accurately measure satisfaction and impact on the perception of service excellence received, compared with the service expected.^21^ The Service Performance (SERVPERF) measurement is a modified version of the SERVQUAL measurement and is based on the perception component alone, aiming to equip service organisations to recognise service weaknesses and then implement improvement strategies.^22^ Rodrigues et al., who had evaluated the similarities and differences of SERVQUAL and SERVPERF, concluded that both measures have to be performed for meaningful conclusions on service quality.^23^ However, Andronikidis and Bellou and Shafei et al. concluded that both measures displayed legitimacy and consistency and can be used to measure service quality.^22,24^ In this study, an adapted SERVPERF questionnaire was used because the model provided a higher correlation between variables and therefore had higher discriminant validity.^25^ The frequent use of the SERVPERF framework indicates that consensus has been reached in terms of its value and application in service quality measurement.^21,26^

The adapted SERVPERF questionnaire consisted of 20 questions evaluating the respondent’s perception of the quality of service received.^27^ The questions were sectioned into the five dimensions of service quality, namely tangibles, responsiveness, reliability, assurance and empathy. Service quality was evaluated on a Likert scale from 1 to 7, in terms of the respondent’s perception of the service experienced. Each respondent answered the same set of questions, allowing for an efficient way to collect data from a large sample and facilitating structured data analysis. Employing a 7-point Likert scale ensured the sensitivity of the questionnaire, giving the respondents the option to indicate to which degree they agree with a statement.^28^ Questionnaires were distributed in hard copy to respondents at each clinic. The purpose of the survey was explained to potential respondents, and if they were willing to participate, they self-administered the questionnaire.

### Data analysis

Data input, processing and analysis were completed by Excel (2016) and a combination of Statistica (13.5) and R lmer package. Data were analysed using descriptive, bivariate and multivariate statistical analyses. Descriptive analysis included mean and standard deviation calculations on respondents’ demographics and perceptions of the quality of service received. Analysis of variance (ANOVA) was performed to compare perceptions within each dimension to determine the most significant gaps in service quality. The *p*-values of less than 0.05 were considered to be statistically significant. The internal consistency of the questions relating to the perception of service was tested by applying Cronbach’s criterion. The Cronbach’s coefficient of 0.83 indicated that the sample is representative of the population.^28,29^ Moreover, exploratory factor analysis tested and confirmed the underlying constructs of service quality. This enabled an analysis of the interrelationship between the dimensions.

### Ethical considerations

Ethical approval to conduct the study was obtained from the Research Ethics Committee for Social, Behavioural and Education Research (REC:SBE) and informed consent was obtained from the respondents (ref. no. REC-2020-15459).

## Results

The questionnaire was administered across three mobile healthcare clinics, and 111 responses were collected. Of the 111 responses received, only 92 respondents completed their gender field and 103 respondents completed the age field on the questionnaire.

**FIGURE 1:**
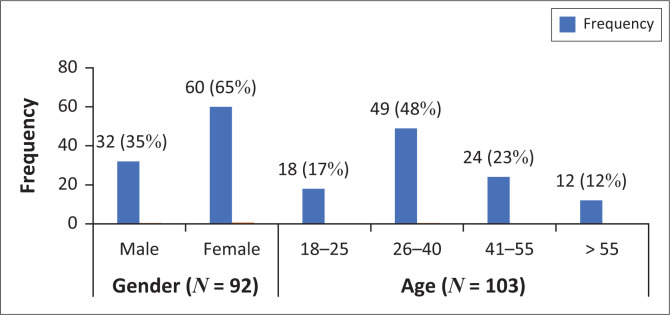
Age and gender distributions.

The age and gender distributions amongst the respondents are illustrated in Figure 1, which depicts that approximately 50% more female respondents visited the clinics during that time, and people in the 26–40 years age group used the clinic service more than any of the other age groups.

The primary objective of the study was to measure the current level of service quality at different clinics from the perspective of the patient. According to the ANOVA in Table 1, most respondents scored close to the upper limit of the 7-point Likert scale, with minimal variance in the data. There was a statistically significant difference in the mean scores of the dimensions as the *p*-value is less than 0.05, (*F* [4.430] = 3.97, *p* < 0.01). The observed difference was between the tangible dimension and the rest of the dimensions. The *p*-values of the responsiveness, reliability assurance and empathy dimensions are all above 0.05, as depicted in Table 1, thereby accepting the hypothesis that the means of the dimensions are equal. There were therefore no differences detected in the perception of service quality with regard to these dimensions, and the score was close to the upper limit.

**TABLE 1:** *Post hoc* results of analysis of variance between dimensions.

Dimension	Tangibles	Responsiveness	Reliability	Assurance	Empathy
Tangibles	-	< 0.01	0.02	< 0.01	< 0.01
Responsiveness	< 0.01	-	0.72	0.70	0.34
Reliability	0.02	0.72	-	0.46	0.19
Assurance	< 0.01	0.70	0.46	-	-
Empathy	< 0.01	0.34	0.19	0.58	-

**TABLE 2:** Analysis of variance results per dimension across gender and age groups.

Dimension	*F*	*p*
**Tangibles**		
Age	1.34	0.27
Age correlation	1.03	0.42
Gender	0.21	0.65
Gender correlation	0.51	0.67
**Responsiveness**		
Age	3.73	0.01
Age correlation	1.11	0.36
Gender	10.02	< 0.01
Gender correlation	0.89	0.41
**Reliability**		
Age	2.20	0.09
Age correlation	1.16	0.31
Gender	6.33	0.01
Gender correlation	1.53	0.19
**Assurance**		
Age	2.03	0.11
Age correlation	1.67	0.09
Gender	0.64	0.42
Gender correlation	0.44	0.72
**Empathy**		
Age	0.96	0.42
Age correlation	1.14	0.34
Gender	2.23	0.14
Gender correlation	1.00	0.39

The second objective of the study was to identify any gaps where service quality improvement can have a positive effect on the perceived value for the patients. As the overall results depicted a perception of excellent service quality, additional analysis was conducted to ascertain whether age or gender had an influence on the responses and whether there were any differences observed in responses between the clinics. The analysis included an investigation per dimension and an investigation whether the questions per dimension had significant differences per response. No differences were detected in the perceptions of service quality amongst the different clinics (*p* = 0.7). All individual *p*-values were greater than 0.05; therefore, there were no differences detected amongst the answers of the dimensions and individual questions per dimension across the different clinics.

An ANOVA per gender shows that both genders scored perceived service quality between 6.6 and 7.0 on the Likert scale. Although there was a statistically significant difference (*p* = 0.05) between the scores of the genders for the responsiveness and reliability dimensions, these differences were smallg as both genders perceived service quality to be excellent, with scores between 6.5 and 7.0 on the Likert scale.

The *p*-value (< 0.01) is less than 0.05; therefore, the genders did not score the questions in this dimension equally. It was evident that question 4 in the tangibles dimension was scored differently across the genders. In question 4, ‘All brochures and advertisements at the healthcare clinic are visually appealing’, this question was scored differently across genders. However, the question was still scored close to the upper limit of the questionnaire.

Age groups 18–25 years and 26–40 years perceived service quality as slightly lower than the older groups. Although the younger age ranges perceived marginally lower service quality than the older age ranges (*p* = 0.03), the difference was not statistically significant, and all age groups perceived service quality to be excellent, with scores between 6.5 and 7.0 on the Likert scale.

## Discussion

In general, the results reflected that the respondents were very satisfied with the service quality experienced at the three clinics. This perception of higher service quality could be because of various factors, including an emphasis on service quality by the funders of the project. The results revealed that the tangible dimension scored slightly lower than the other dimensions. This result is similar to a previous study that found assurance and responsiveness as the more important dimensions and tangibles as the least important dimension.^29^ Akdere et al. further suggested that augmenting the visual attractiveness and cleanliness of the buildings could increase the perception of the tangibles dimension.^29^ However, in this study the score was already very high, and the Cohen’s analysis revealed only small differences between tangibles and the rest of the dimensions.

The demographic representation of gender amongst the respondents supports the inference that women use medical services more than men, and their perception of service quality is therefore influenced by their different behaviours and beliefs.^30^ The results of this study suggested that male service recipients regarded responsiveness and reliability marginally higher than the female recipients, thereby supporting the view of Abu-Salim et al. that male patients are more rational and time-conscious in their perception of service quality.^30^

The findings of this study indicate that the five dimensions within the SERVPERF questionnaire do predict overall service quality; however, as most respondents scored close to the upper limit of the questionnaire, with minimal variance in the data, the results could also have been subjected to several biases.^31^ The biases included response bias, where respondents are completing the questionnaire without reading the questions, and extreme bias, with most of the scores close to the upper limit of the scale.

Respondents might also have perceived that lower scoring could result in the clinic service being terminated, even though the purpose and anonymity of the questionnaire had been communicated and guaranteed. The perception of service quality experienced by respondents could also have been influenced by comparison to the other available health services. The alternative for service recipients is often government services with a long waiting time and inadequate supplies.

### Limitations

Although the study revealed an excellent perception of service quality from the patients’ perspective, the absence of an adequate comparator organisation can be listed as a limitation. In addition, more insight is required into nonmonetary sacrifice and perception of service quality from a patient’s perspective. Furthermore, more empirical studies should be conducted to confirm the usefulness of the SERVPERF scale in terms of its reliability and validity in a NPO setting.

### Recommendations

Future research recommendations include a wider population sample by including more mobile clinics from different organisations to confirm the validity and reliability of solicited service quality questionnaires in a NPO setting. In addition, the SERVQUAL scale can be applied to measure whether there are any gaps in the service expected and the service received. Furthermore, the SERVPERF scale applied in this research study measured all dimensions as equal; however, the dimensions can be weighted to ascertain whether any dimension is perceived as more or less important when service quality is being assessed. Although service quality measured at the clinics was very high, the current standard service quality measure for a profit setting might not be optimal in an NPO setting. The minimal variance in the data suggest that additional research is necessary into what would matter for the patient in an NPO setting, such as what their baseline expectations and their alternatives are.

## Conclusion

The application of a modified SERVPERF questionnaire has revealed a favourable result in this study with a high level of service quality and customer satisfaction. The results, however, might have been influenced by comparing services of alternative health institutions. Nevertheless, healthcare service organisations cannot afford to ignore the needs and expectations of their service recipients. As context matters, different service settings have to be taken in account. Organisations therefore have to engage in continuous measurement and improvement of their service quality as part of their quality management process.^27^ Not-for-profit organisations should strive towards maintaining high standards and avoid complacency by opening various channels for patient feedback and conducting regular customer feedback surveys.
